# Clinical efficacy of intravitreal aflibercept injection versus vitrectomy with panretinal photocoagulation for patients with proliferative diabetic retinopathy: A protocol for systematic review and meta-analysis

**DOI:** 10.1097/MD.0000000000025354

**Published:** 2021-04-09

**Authors:** Yao Hu, Jinxia Shen, Yi Peng

**Affiliations:** Department of Ophthalmology, Wuhan Eyegood Ophthalmic, Wuhan, China.

**Keywords:** intravitreal aflibercept injection, meta-analysis, panretinal photocoagulation, proliferative diabetic retinopathy, protocol, vitrectomy

## Abstract

**Background:**

In the current literature, it is still controversial whether intravitreal aflibercept injection can provide better vision restoration compared with vitrectomy with panretinal photocoagulation (PRP) for proliferative diabetic retinopathy (PDR) patients. Given that there is no high-quality meta-analysis or review to incorporate existing evidence, the purpose of this study is to systematically review the level I evidence in the literature to ascertain whether intravitreal aflibercept injection can provide better vision restoration compared with vitrectomy with PRP for PDR patients.

**Methods:**

The systematic literature review is structured to adhere to PRISMA guidelines (Preferred Reporting Items for Systematic Reviews and Meta-analyses), which include requirements deemed essential for the transparent reporting of results. A systematic search will be performed in Web of Science, Embase, Scopus, Science Direct, Cochrane Library up to and inclusive of March 19, 2021. The method of data extraction will follow the approach outlined by the Cochrane Handbook for Systematic Reviews of Interventions. The primary outcome is change in best-corrected visual acuity. The secondary outcomes are change in area of neovascularization and change in area of retinal nonperfusion. Where disagreement occurs, this will be resolved through discussion. All outcomes are pooled on random-effect model. A *P* value of < .05 is considered to be statistically significant.

**Results:**

The results of our review will be reported strictly following the PRISMA criteria.

**Conclusions:**

The hypothesis of the study was that visual acuity recovery would be faster with vitrectomy because the blood is mechanically cleared during surgery.

**Registration number:**

10.17605/OSF.IO/NCAXW.

## 1 Introduction

Proliferative diabetic retinopathy (PDR) is the most common cause of severe vision loss in diabetic patients. If left untreated, serious complications can occur, including vitreous hemorrhage, retinal detachment, and severe vision loss.^[1]^ Since the 1970s, vitrectomy has become the standard treatment for opaque vitreous hemorrhage. Removal of the vitreous gel during surgery quickly clears the hemorrhage, removing the traction of new blood vessels that cause new vitreous hemorrhage, and combining with intraoperative panretinal photocoagulation (PRP) to treat new blood vessel formation.^[2,3]^ Although surgical techniques have improved over the past 50 years, the risk of complications remains. In addition, PRP may be associated with serious side effects, including peripheral vision loss, nyctalopia, development of macular edema or worsening of previous edema, and damage to the posterior ciliary nerve, resulting in pupil dilation, and loss of adaptation. Therefore, clinicians are highly interested in developing nonsurgical approaches.^[4,5]^

Recent intravitreal therapies targeting vascular endothelial growth factor, such as pegaptanib, ranibizumab, bevacizumab, and aflibercept, have introduced a paradigm shift in the management of a wide array of ocular diseases, including neovascular age-related macular degeneration, diabetic macular oedema, and retinal vein occlusions.^[6–8]^ Antivascular endothelial growth factor treatment has superseded macular laser treatment and is now the standard of care in patients with center-involving diabetic macular edema. However, therapeutic options for PDR remain limited to PRP despite several clinical and preclinical studies indicating that vascular endothelial growth factor is the key causative factor of retinal neovascularisation.^[9]^ Recent evidence also indicates that monthly antivascular endothelial growth factor treatment can reduce the severity of and delay the progression of diabetic retinopathy over 24 months.^[10]^

In the current literature, it is still controversial whether intravitreal aflibercept injection can provide better vision restoration compared with vitrectomy with PRP for PDR patients. Recent randomized cohort studies have tried to resolve this issue, but have reached inconsistent conclusions.^[11–14]^ Given that there is no high-quality meta-analysis or review to incorporate existing evidence, the purpose of this study is to systematically review the level I evidence in the literature to ascertain whether intravitreal aflibercept injection can provide better vision restoration compared with vitrectomy with PRP for PDR patients. The hypothesis of the study was that visual acuity recovery would be faster with vitrectomy because the blood is mechanically cleared during surgery.

## 2 Materials and methods

### 2.1 Registration

The systematic review protocol has been registered on Open Science Framework registries. The registration number is 10.17605/OSF.IO/NCAXW. We will update our protocol for any changes in the entire research process if needed. Ethical approval is not necessary because the present meta-analysis will be performed based on previously published studies.

### 2.2 Literature search

**Figure 1:**
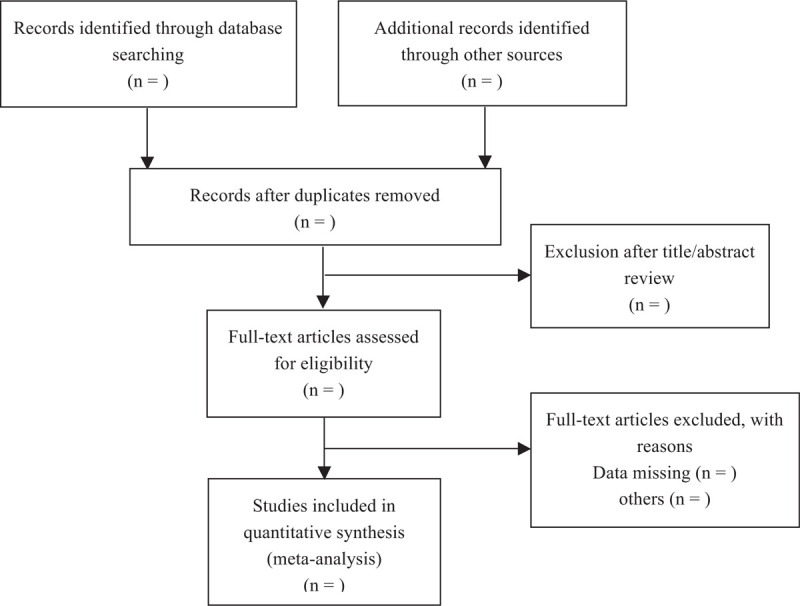
Flow diagram of study identification.

The systematic review and meta-analysis will be conducted according to the Preferred Reporting Items for Systematic Reviews and Meta-Analyses statement. A systematic search will be performed in Web of Science, Embase, Scopus, Science Direct, Cochrane Library using the following search strategy up to and inclusive of March 19, 2021: (proliferative diabetic retinopathy OR PDR) AND (aflibercept) AND (photocoagulation OR PRP) AND (vitrectomy) AND (random OR prospective OR blind). There are no language restrictions. Subsequently, an additional search will be performed in PubMed using the same search terms (Fig. 1).

### 2.3 Eligibility criteria

Study included in this review has to meet all of the following inclusion criteria in the PICOS order: population: patients with proliferative diabetic retinopathy; intervention group (group 1): intravitreal aflibercept injection; comparison group (group 2): vitrectomy with panretinal photocoagulation; outcome measures: at least one of the following outcome measures was reported: change in best-corrected visual acuity, change in area of neovascularization, and change in area of retinal nonperfusion; study design: level I randomized controlled trials. Biomechanical studies, in vitro studies, review articles, surgical techniques, case reports, letters to the editor, and editorials are excluded. Prospective nonrandomized studies and retrospective studies are also excluded.

### 2.4 Study selection

The first author conducts a preliminary screening based on the title to eliminate any research not related to the topic. A log of excluded studies is kept with the rationale for exclusion. Subsequently, all remaining abstracts are reviewed by the primary author, and the selection criteria are applied. Studies identified for full text review are evaluated by 2 authors for inclusion in the study. Disagreements are resolved through a discussion with a third review author. Journal titles and authors’ names are not glossed over in the research selection process. A manual search of the bibliographies of included studies is performed to ensure that the overall search is comprehensive and complete.

### 2.5 Data extraction

The method of data extraction will follow the approach outlined by the Cochrane Handbook for Systematic Reviews of Interventions. Two independent authors extract the following descriptive raw information from the selected studies: study characteristics such as the first author, publication year, study design, follow-up period; patient demographic details such as patients’ number, average age, and gender ratio. The primary outcome is change in best-corrected visual acuity. The secondary outcomes are change in area of neovascularization and change in area of retinal nonperfusion. Where disagreement in the collection of data occurs, this will be resolved through discussion. The corresponding author will be contacted and asked to provide the data if the standard deviation (SD) is not reported. In the case of no response, the SD is calculated from the available data according to the previously validated formula: (higher range value − lower range value)/4 or interquartile range/1.35. The highest SD is used if the SD cannot be calculated using this approach. If necessary, we will abandon the extraction of incomplete data.

### 2.6 Statistical analysis

Review Manager software (v 5.3; Cochrane Collaboration) is used for the meta-analysis. Extracted data are entered into Review Manager by the first independent author and checked by the second independent author. Risk ratio with a 95% confidence interval or standardized mean difference with 95% CI is assessed for dichotomous outcomes or continuous outcomes, respectively. The heterogeneity is assessed by using the Q test and I^2^ statistic. An I^2^ value of < 25% is chosen to represent low heterogeneity and an I^2^ value of > 75% to indicate high heterogeneity. All outcomes are pooled on random-effect model. A *P* value of < .05 is considered to be statistically significant.

### 2.7 Quality assessment

Each paper is reviewed by 1 reviewer and verified by a second and disagreements are resolved by discussion with a third reviewer. A meta-analysis is conducted when 3 or more trials reported an outcome of interest. Subgroup analyses are planned based on different follow-up periods and the status of the pain assessment. We also perform the sensitivity analysis to evaluate whether the differences of study design have an impact on the overall estimate and data. Furthermore, we do not evaluate the publication bias domain, as the recommendation is not to assess funnel plot asymmetry with meta-analyses of less than 10 trials.

## 3 Discussion

PDR is the commonest cause of severe visual loss in people with diabetes. In the current literature, it is still controversial whether intravitreal aflibercept injection can provide better vision restoration compared with vitrectomy with PRP for PDR patients. Recent randomized cohort studies have tried to resolve this issue, but have reached inconsistent conclusions.^[11–14]^ Given that there is no high-quality meta-analysis or review to incorporate existing evidence, the purpose of this study is to systematically review the level I evidence in the literature to ascertain whether intravitreal aflibercept injection can provide better vision restoration compared with vitrectomy with PRP for PDR patients. The hypothesis of the study was that visual acuity recovery would be faster with vitrectomy because the blood is mechanically cleared during surgery.

## Author contributions

**Conceptualization:** Yao Hu, Jinxia Shen.

**Data curation:** Yao Hu, Jinxia Shen.

**Formal analysis:** Yao Hu, Jinxia Shen.

**Funding acquisition:** Yi Peng.

**Investigation:** Yao Hu, Jinxia Shen.

**Methodology:** Yao Hu, Jinxia Shen, Yi Peng.

**Project administration:** Yi Peng.

**Resources:** Yi Peng.

**Software:** Yao Hu.

**Supervision:** Yi Peng.

**Writing – original draft:** Yao Hu, Jinxia Shen.

**Writing – review & editing:** Yi Peng.
